# S-1 monotherapy versus S-1 combination therapy in gemcitabine-refractory advanced pancreatic cancer: A meta-analysis (PRISMA) of randomized control trials

**DOI:** 10.1097/MD.0000000000007611

**Published:** 2017-07-28

**Authors:** Sheng Zhong, Shuai Qie, Liu Yang, Qi Yan, Linna Ge, Zhongfeng Wang

**Affiliations:** a Department of Neurosurgery, the First Hospital of Jilin University; b Clinical College, Jilin University, Changchun, China; c Department of the Radiotherapy, Hebei University Affiliated Hospital, Baoding; d Public Health College, Jilin University; e Basic Medical College, Qiqihar Medical University, Qiqihar; f Radiology Department, Jixi Mining General Hospital, Jixi; g Hepatopancreatobiliary Medicine Department, the First Hospital of Jilin University, Changchun, China.

**Keywords:** advanced pancreatic cancer, combination therapy, irinotecan, leucovorin, monotherapy, oxaliplatin, S-1, second-line chemotherapy

## Abstract

**Background:**

Pancreatic cancer (PC) is one of the most lethal digestive system tumors. Most new cases are diagnosed based on metastasis or local aggression and are known as “advanced PC.” Recently, studies investigating S-1 have indicated that it has a better clinical curative effect on PC. We conducted a meta-analysis to evaluate the efficacy and safety of S-1 monotherapy compared with S-1 combination regimens in patients with gemcitabine (GEM)-refractory PC.

**Methods:**

Trials published between 1978 and 2016 were identified by an electronic search of public databases (Medline, Embase, and the Cochrane Library). All prospective studies were independently identified by 2 authors for inclusion. The response rate (RR), progression-free and overall survival (PFS and OS, respectively), and the primary toxicities were extracted for the meta-analysis.

**Results:**

Four randomized controlled trials consisting of 623 patients were included in the analysis, among which 315 patients underwent S-1 monotherapy and 308 patients underwent S-1 combination therapy. The pooled data showed a significantly higher response rate and longer PFS in the S-1 combination group than in the S-1 monotherapy group (RR, 1.75; 95% confidence interval [CI], 1.19–2.57; *P* = .005 and hazard ration [HR], 0.75; 95% CI, 0.62–0.91; *P* = .005). There were no significant differences in OS or adverse events.

**Conclusions:**

Compared with the S-1 monotherapy group, the S-1 combination group had a higher response rate and longer PFS. Both groups had few adverse events, which were balanced between the groups. The subgroup analysis suggested that S-1 combination regimens with leucovorin or irinotecan (CPT-11) provided promising efficacy. These promising combination regimens should be considered for patients with advanced PC who choose S-1 as their second-line therapy.

## Abstract 2

Supplemental Digital Content is available in the text

## 1 Introduction

Pancreatic cancer (PC) is known for its extremely poor prognosis and is one of the most lethal digestive system tumors.^[1]^ According to the latest cancer data released by the American Cancer Society, the 5-year survival rate of PC patients is <5%, and the median survival time is 2 to 4 months. PC is the fourth leading cause of cancer-related mortality worldwide. Although great progress has been achieved in imaging diagnostic modalities such as endoscopic ultrasonography and magnetic resonance imaging,^[2]^ PC is still a high-risk tumor with a poor prognosis. Almost 80% of new cases are diagnosed based on metastasis or local aggression and are known as “advanced PC.”^[3]^ Unfortunately, these patients have missed their best opportunity to undergo tumor resection removal, which is the most effective method to cure PC. Hence, there is an urgent need to explore high-efficacy chemotherapeutic regimens to improve the prognosis of patients with advanced PC. Several standard therapeutic regimens for advanced PC have been developed to date. Gemcitabine (GEM) is currently used as the first-line chemotherapeutic agent and has been used as the standard treatment for advanced PC since the 1900s. Although GEM can significantly prolong overall survival (OS), its therapeutic efficacy for improving the long-term prognosis of PC remains limited, with a median survival of <6 months.^[4]^ FOLFIRINOX (5-fluorouracil, leucovorin, irinotecan, and oxaliplatin combination) recently becomes a standard regimen for gemcitabine-refractory pancreatic cancer. However, it could only be administrated to patients who have good medical conditions because of its severe side effects and toxicity.^[5]^ CONKO-003 trial demonstrates that oxaliplatin, leucovorin (LV), and fluorouracil (OFF) regime significantly improve patients’ survival compared with fluorouracil plus LV (FF) in advanced PC,^[6]^ but their use appears to have certain limitations, and the regimens must be improved prior to their broad application. Thus, to improve the prognosis of unresectable PC, the development of a new and effective therapeutic regimen is essential, and more effective and safe chemotherapeutic treatments are required.

S-1 is an oral agent that consists of tegafur, 5-chloro-2, 4-dihydroxypyridine, and potassium oxonate at a molar ratio of 1:0.4:1 and was demonstrated to have a potential therapeutic effect for PC in many recent second-line chemotherapy studies.^[7–10]^ S-1 is an oral fluoropyrimidine derivative that was designed to improve the antitumor activity of 5-FU while reducing its gastrointestinal toxicity.^[11]^ Recent studies investigating S-1 have demonstrated that it has a better clinical curative effect not only for gastric carcinoma but also for head-neck tumors, non-small cell lung cancer (NSCLC), and PC. Although several studies have focused on the therapeutic efficacy and safety of S-1 monotherapy versus S-1 combination therapy, these studies have not provided a definitive conclusion. Additionally, various and controversial types of chemotherapeutic agents have been combined with S-1. S-1 has been combined with irinotecan (CPT-11), oxaliplatin, or leucovorin alone in previous studies.^[7–10]^ However, which combination regimen (or S-1 monotherapy) can achieve the best curative effect still remains unclear. Here, we conducted a meta-analysis to evaluate the efficacy and safety of S-1 monotherapy compared with S-1 combination regimens in patients with GEM-refractory PC in a second-line setting. Identification of the therapeutic efficacy of different regimens will provide guidelines and help guide therapeutic decisions for advanced PC.

## 2 Methods and materials

This study was approved by the Ethics Committee of First Hospital of Jilin University. The following steps were performed in the present analysis: definition of the outcomes (definition of the question the analysis was designed to answer), definition of the criteria applied for the selection of eligible trials, definition of the search strategy, and detailed description of the statistical method.

### 2.1 Outcome definition

Two investigators independently extracted the data from each eligible study. We considered studies that contained only the S-1 agent as the S-1 monotherapy group and those that consisted of S-1 combined with other chemotherapeutic drugs as the S-1 combination group. We recorded the following information: name of the first author, year of publication, PS scores of cases in the 2 groups, histology, sex ratio, chemotherapeutic regimens, and number of cases in the 2 groups. We also recorded the response rate, the hazard ratios for overall survival (OS) and progression-free survival (PFS) in patients in the different groups, and the primary related adverse effects. The OS outcome was defined as the time between randomization and death due to any cause or the date of the last follow-up visit for surviving cases. The PFS outcome was defined as the time between randomization and disease progression, death without progression, or the date of the last follow-up visit for surviving patients without progression.

### 2.2 Search strategy

Our search strategy was described in detail in Appendix 1. We searched the PubMed (Medline) and Embase (Ovid) databases for articles published between January 1996 and September 2016 as well as the Cochrane Library and Web of Science. We used a sensitive search strategy with keywords related to “advanced pancreatic cancer,” “combination therapy,” “S-1,” “randomized controlled trial,” and “controlled clinical trial.” The article language was restricted to English. All relevant references as well as all additional studies of potential interest were scanned carefully by 2 authors of this article (ZS and QS). They analyzed and selected all eligible studies independently.

### 2.3 Inclusion and exclusion criteria

We gathered all phase II–III and prospective and randomized trials that were published as formal papers. All articles that met the following criteria were eligible:

About patients: patients with a histologically or cytologically confirmed diagnosis of pancreas adenocarcinoma or adenosquamous carcinoma which was refractory to gemcitabine treatment; patients that did not undergo prior treatment, including surgery, chemotherapy, or radiation therapy; patients without chemotherapy contraindications or serious vital organ dysfunction and performance status (PS) scores of 0 to 1.

About study design and comparison: randomized controlled trial (RCT) comparing S-1 monotherapy with S-1 combination therapy in all age groups.

About outcome measurements: analyses that included the response rate, overall survival (OS), and progression-free survival (PFS) with 95% confidence intervals (CIs) or relevant data, as well as adverse events (grade ≥3) or hematological toxicity (grade ≥3); a response rate should be determined by the response evaluation criteria in solid tumors (RECIST) or WHO evaluation criteria. A tumor completely disappeared for more than 4 weeks without any new lesions formation was defined as complete remission (CR). A tumor regressed ≥50% for more than 4 weeks without development of any new carcinous lesions was defined as partial response (PR). Both CR and PR were defined as responding when calculating response ratio (RR). The sum of the longest diameters (LD) of the target lesions increased >25% compared with the initiation of trials or there are 1 or more new carcinous lesions formation was defined as progressive disease (PD). Tumor regressed ≤50% or increased ≤25% was defined as stabilized disease (SD). Toxicity was assessed based on the National Cancer Institute Common Terminology Criteria regarding to adverse events. Studies meeting any 1 of the following criteria were excluded: laboratory studies, letters, review articles, low quality clinical control study, or case reports; animal experimental studies; the outcomes of interest (i.e., OS and RR) were impossible to calculate or the standard deviations and CIs of the tested parameters were not reported; and an absence of key information such as the sample size, HR, 95% CI, and *P* value.

### 2.4 Study selection

The eligibility selection was first conducted by screening titles, key words, and abstracts, followed by perusing the full text of the articles. Selecting all studies was conducted independently by 2 reviewers according to the inclusion and exclusion criteria. A third reviewer was invited to determine when there were disagreements on whether an article should be included.

### 2.5 Data extraction

Two authors independently extracted data from the 4 eligible studies. When the extracted data were not uniform, a third reviewer was needed to make a final determination. The following data of all eligible trials were extracted: name of the first author, trial phase, publication year, type of study, number of enrolled patients, sex ratio, average ages, patients’ performance status, interventions, and outcomes.

### 2.6 Quality assessment

Cochrane handbook was used to evaluate the quality of the trials. The following items were carefully assessed and recorded: blinding; allocation concealment; method of randomization; exclusion from the analysis by arm; patient follow-up time in each group; and number of patients lost to follow-up in each group. The assessment outcome was shown in Appendix 2.

### 2.7 Statistical analysis

Statistical analyses were performed using the Revman 5.2 Chi-square and *I*^2^ tests to assess the heterogeneity among the different studies. *P* > .1 and *I*^2^ < 50% in the q test was considered as there is no heterogeneity, and a fixed-effect model was applied to pool these study results. Heterogeneity was considered as significant when *P* ≤ .1 and *I*^2^ ≥ 50%. In this case, the random-effect statistical model was used.

## 3 Results

### 3.1 Study identification and selection

**Figure 1:**
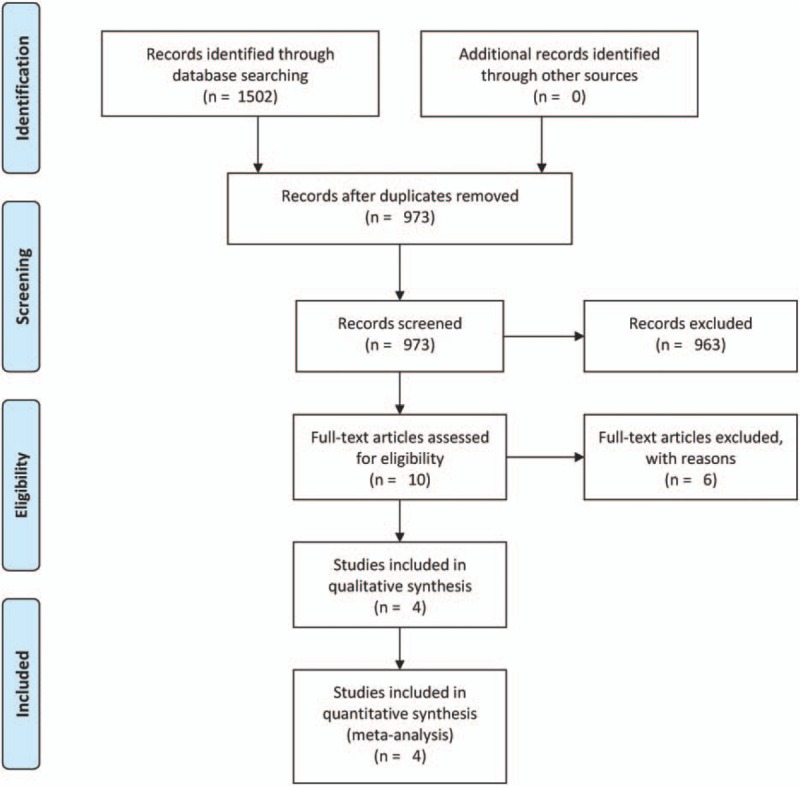
Flowchart of the study selection.

Using the outlined search strategy, a total of 1502 citations were obtained for the title and abstract review. Of the 1502 citations, 963 were not relevant and 529 were duplicates. The full texts of the remaining 10 studies were retrieved for review; 6 of the 10 full-text articles were excluded (as shown in Fig. 1).

### 3.2 General characteristics of the included studies

The characteristics of the included studies are shown in Table 1. All included studies were phase II RCTs that compared the therapeutic efficacy and safety of S-1 monotherapy and S-1 combination therapy. A total of 623 patients were included in the analysis, with 315 patients undergoing S-1 monotherapy and 308 patients undergoing S-1 combination therapy. All studies were conducted in Japan and China. The ages of the patients varied from 25 to 85 years. The therapeutic efficacy outcomes included the response rate, OS and PFS, and adverse events such as neutropenia, diarrhea, and nausea. The studies conducted by Ueno and Ge were phase II randomized comparisons of S-1 monotherapy with S-1 plus leucovorin combination therapy. A slight difference in the dosage and usage of leucovorin was noted. In Ueno (2016),^[7]^ leucovorin was administered at a fixed 25 mg dose with each S-1 dose. In that study, patients assigned to the S-1 leucovorin combination group received S-1 plus LV orally for 1 week, followed by a 1-week rest period; this regimen was repeated every 2 weeks. In the Ge study, leucovorin was administered at a dose of 25 mg as reported in Ueno (2016);^[7]^ however, leucovorin was only combined with S-1 from days 1 to 7 in a 3-week cycle. Thus, the total dose of leucovorin in the Ge study was approximately one-third of that in the Ueno (2016)^[7]^ study. The Mizuno (2013)^[10]^ study was a phase II randomized comparison of S-1 monotherapy with S-1 plus CPT-11 combination therapy. The Ohkawa study was a phase II randomized comparison of S-1 monotherapy with S-1 plus oxaliplatin combination therapy. The characteristics of the included studies are summarized in Table 1.

**Table 1:**
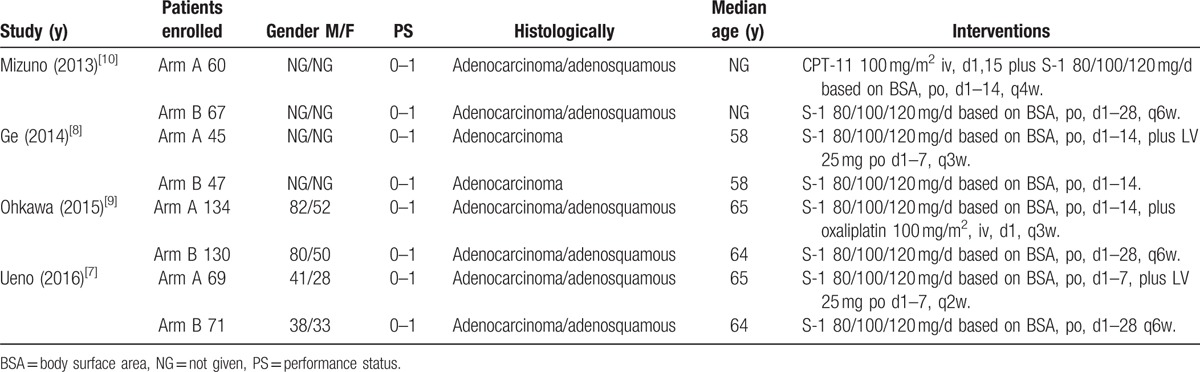
Characteristics of the studies included in the meta-analysis.

### 3.3 Meta-analysis results

#### 3.3.1 Response rate

**Figure 2:**
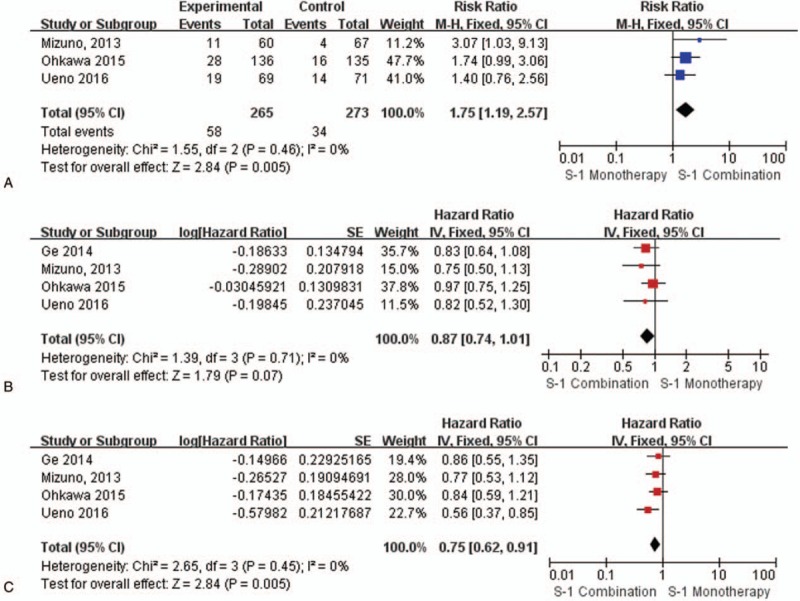
A. Forest plot of the response rate. There was a significant difference between the 2 arms (RR, 1.75; 95% CI, 1.19–2.57; *P* = .005). B. Forest plot of OS. There was no significant difference between the 2 arms (HR, 0.87; 95% CI, 0.74–1.01; *P* = .07). C. Forest plot of PFS. There was a significant difference between the 2 arms (HR, 0.75; 95% CI, 0.62–0.91; *P* = .005). CI = confidence interval, HR = hazard ration, PFS = progression-free survival, RR = response rate.

The objective response rate (RR) was included in 3 trials. There was no heterogeneity (*P* = .46, *I*^2^ = 0%) among the 3 studies. Thus, the fixed effect model was used for the meta-analysis. The meta-analysis revealed a significant difference in the response rate between the 2 groups, the S-1 combination group had a higher response rate than the S-1 monotherapy group (RR, 1.75; 95% CI, 1.19–2.57; *P* = .005) (Fig. 2A).

#### 3.3.2 Overall survival

Each of the included trials reported OS data. The heterogeneity analysis provided an *I*^2^ value that was equal to 0% (*P* = .71), which demonstrated no statistical heterogeneity. Thus, a fixed-effect model was employed for the analysis. There was no significant difference in overall survival between the S-1 monotherapy and S-1 combination groups (HR, 0.87; 95% CI, 0.74–1.01; *P* = .07) (Fig. 2B). Compared with S-1 monotherapy, S-1 combination groups cannot prolong OS in PC patients.

#### 3.3.3 Progression-free survival

All 4 trials reported PFS. The results showed no heterogeneity among these trials (*I*^2^ = 0%, *P* = .45), and thus a fixed-effect model was employed for the analysis. There were significant differences in PFS between the 2 groups. PFS was significantly longer in the S-1 combination group than in the S-1 monotherapy group, and the S-1 combination group exhibited prolonged PFS compared with the S-1 monotherapy group (HR, 0.75; 95% CI, 0.62–0.91; *P* = .005) **(**Fig. 2C).

#### 3.3.4 Adverse events

**Figure 3:**
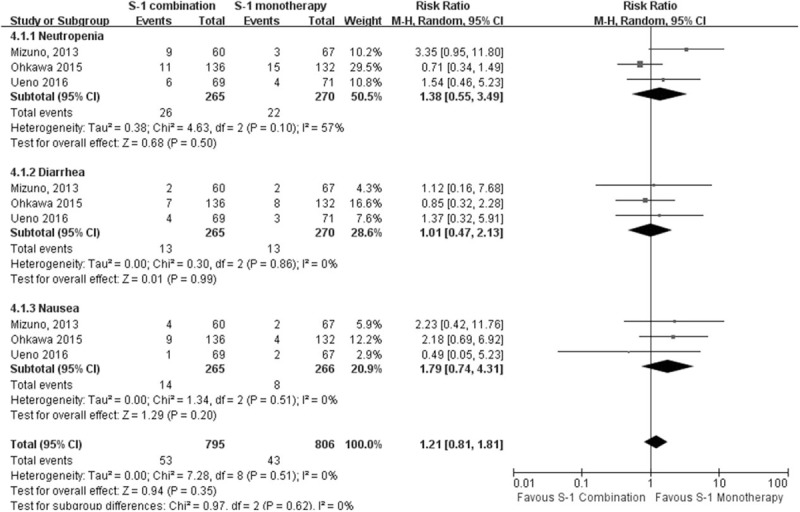
Forest plot of adverse events. There were no significant differences between the 2 arms in terms of neutropenia, diarrhea or nausea.

Three studies reported the occurrence of drug-related adverse events (grade ≥3) such as neutropenia, diarrhea, and nausea. A random effects model was used for all groups. The results demonstrated the incidence of neutropenia (RR, 1.38; 95% CI, 0.55–3.49; *P* = .50), diarrhea (RR, 1.01; 95% CI, 0.47–2.13; *P* = .99), and nausea (RR, 1.79; 95% CI, 0.74–4.31; *P* = .20). These results implied that there were no differences between the groups, suggesting that both therapeutic regimens were well tolerated. (As shown in Fig. 3)

#### 3.3.5 Subgroup analysis

**Figure 4:**
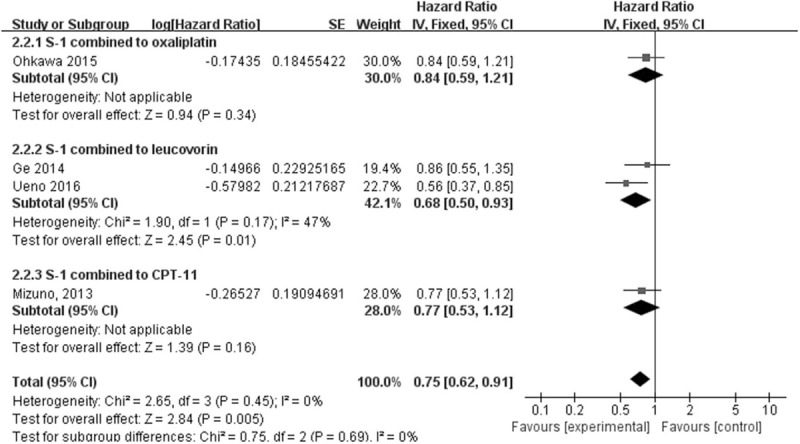
Forest plot of PFS in the subgroup analysis.

We also performed a subgroup analysis stratified by the type of regimen to identify different effects. There were no significant differences in OS. None of the therapeutic regimens resulted in a significantly longer OS, although the OS in the S-1 combination group appeared to be prolonged. For PFS, S-1 combined with leucovorin (HR, 0.68; 95% CI 0.50–0.93) had a better effect than the other 2 regimens. There was no obvious improvement regarding PFS in S-1 combined with oxaliplatin or CPT-11, indicating that the robust effect of S-1 combined with leucovorin led to a longer PFS in the S-1 combination group (HR, 0.75; 95% CI 0.62–0.91). (As shown in Fig. 4) For RR, S-1 combined with the CPT-11 regimen led to significantly higher response rate than S-1 monotherapy (RR, 3.07; 95% CI 1.03–9.13). The other 2 regimens also resulted in higher RR compared with S-1 monotherapy, although this difference was not significant. (As shown in Fig. 2A)

#### 3.3.6 Sensitivity analysis

Each study was excluded individually to validate the reliability of the conclusion. There was no change in the significance of the RR, OS, neutropenia, diarrhea, or nausea. Nevertheless, the PFS outcomes changed rapidly after excluding the study by Ueno (2016)^[7]^ (shown in Appendix 3), resulting in a lack of a significant difference between the 2 groups (HR, 0.82; 95% CI 0.56–1.02).

#### 3.3.7 Publication bias

Funnel plots showed there was no publication bias (shown in Appendix 4). However, the number of the original studied included in this meta-analysis was only 4, the accuracy of the analysis result was limited.

## 4 Discussion

Pancreatic cancer (PC) is currently the fourth leading cause of cancer-related mortality worldwide and has an extremely poor prognosis; thus, PC is one of the greatest challenges for oncologists.^[1]^ Upon diagnosis, most patients have metastases or local aggression. These patients miss their golden period for surgical resection. Thus, there is a strong need for effective systemic treatments for PC. Gemcitabine (GEM) has been used as the standard systemic chemotherapeutic agent for advanced PC for many years, although clinical data have demonstrated that GEM combined with S-1 prolongs patient survival over GEM alone.

S-1 is an oral fluoropyrimidine derivative that was designed to improve the antitumor activity of 5-FU while reducing its gastrointestinal toxicity.^[11]^ Recently, studies investigating S-1 have indicated that it has a better clinical curative effect for advanced PC. S-1 was approved for the treatment of PC in Japan in 2006. The Gemcitabine and S-1 Trial (GEST) phase III trial clearly showed that S-1 was not inferior to GEM in terms of the OS rate in patients with metastatic and locally advanced PC in Japan and Taiwan.^[12–15]^ S-1 is currently more commonly used for the treatment of GEM-refractory PC. Many high-quality clinical trials have been performed to investigate the therapeutic efficacy and safety of S-1 combined with multiple chemotherapeutic agents. Ge (2014)^[8]^ and Ueno (2016)^[7]^ both compared the efficacy and safety of oral S-1 as monotherapy or in combination with leucovorin as the second-line treatment for patients with metastatic pancreatic cancer whose disease had progressed on gemcitabine treatment. Ohkawa (2015)^[9]^ compared the therapeutic efficacy of S-1 monotherapy to S-1 combined with oxaliplatin, whereas Mizuno compared the therapeutic efficacy of S-1 monotherapy to S-1 combined with CPT-11. These studies were all included in this meta-analysis.

In this meta-analysis of 4 RCTs including 623 patients, the efficacy and safety of S-1 monotherapy was compared with S-1 combination regimens in patients with GEM-refractory PC. Our results showed that S-1 combination therapy was more effective than S-1 monotherapy, resulting in a significantly higher response rate and longer PFS. Notably, OS was much longer in the S-1 combination group, although this difference was not significant (HR, 0.87, 95% CI 0.74–1.01). The analysis of adverse event outcomes differed slightly between groups, indicating that both therapeutic regimens were well tolerated and modest. In the subgroup analysis, there were no significant differences in OS, and none of the regimens resulted in a significantly longer OS, although it appeared to be longer in the S-1 combination group. For PFS, S-1 combined with leucovorin (HR, 0.68; 95% CI 0.50–0.93) provided the best effect among the 3 regimens. There were no obvious improvements in PFS associated with S-1 combined with oxaliplatin or CPT-11, suggesting that the extraordinary effect of S-1 combined with leucovorin led to longer PFS in the S-1 combination group (HR, 0.75; 95% CI 0.62–0.91). We also noted a certain amount of heterogeneity (*I*^2^ = 47%) in the 2 leucovorin combination groups (Ge, 2014 and Ueno, 2016); this phenomenon might have been caused by the different administration regimens. Ueno (2016)^[7]^ showed that leucovorin was administered at a fixed dose of 25 mg with each S-1 dose. The patients in this study who were assigned to the S-1 and leucovorin combination group received S-1 plus LV orally for 1 week, followed by a 1-week rest; this cycle was repeated every 2 weeks. In the Ge study, leucovorin was administered at a dose of 25 mg as reported by Ueno (2016),^[7]^ but leucovorin was only combined with S-1 from days 1 to 7; subsequently, the patients received S-1 alone from days 7 to 14 in 3-week intervals. Thus, the total dose of leucovorin was approximately one-third of the dose administered in Ueno (2016).^[7]^ This finding suggested that the total dose and usage of adjutant leucovorin might affect the final therapeutic efficacy. Additionally, differences in the baselines and backgrounds of the patients in these 2 studies (Ge, 2014 and Ueno, 2016) might be attributed to the heterogeneity. This result suggested that S-1 combined with leucovorin, as described in Ueno (2016),^[7]^ could effectively prolong patient PFS, provide good efficacy, and good tolerability, and be considered for therapeutic applications. S-1 consists of tegafur, 5-chloro-2, 4-dihydroxypyridine, and potassium oxonate. Tegafur is transferred to 5-fluorouracil by hepatic microsomal cytochrome P450 metabolic enzymes. Leucovorin is a biochemical regulator of 5-fluorouracil; their combination can improve the effectiveness of chemotherapy. Under physiological conditions, the specific underlying mechanism involves the formation of a composite triple complex of deoxy-uridine (dUMP), thymine synthase (TS) and reduced folate provided in vivo (CH_2_FH_4_), which results in the production ofthymidine (dTMP). When 5-fluorouracilis infused, FdUMP is substituted for dUMP and combines with CH_2_FH_4_ and TS to form a triple complex to inhibit TS and halt dTMP generation. CH_2_FH_4_ is present at a lower concentration under physiological conditions, resulting in a weaker suppression effect against TS. The application of exogenous leucovorin into the system can increase the content of CH_2_FH_4_ in the triple composite and enhance the suppression effect on TS and thus the efficiency of 5-fluorouracil.^[16]^

Concerning the RR, S-1 combined with the CPT-11 regimen resulted in a significantly higher RR than S-1 monotherapy (RR, 3.07; 95% CI 1.03–9.13). The other 2 regimens also provided a higher RR compared with the S-1 monotherapy group, although these differences were not significant. This result suggested that S-1 combined with CPT-11 was better than the other regimens in terms of increasing the RR. During the application of a new chemotherapeutic regimen, the safety of the patients is always of paramount importance. This step becomes extremely important when a regimen is applied in patients with poor health who fail first-line therapy. A regimen that causes fewer adverse events can rapidly improve the physical and mental condition of the patient. In this study, we evaluated the safety of various regimens (including neutropenia, diarrhea, and nausea) and demonstrated no significant differences between S-1 monotherapy and combination therapy regimens. This result implied that each combination of S-1 was well tolerated. In the sensitivity analysis, each study was excluded individually to validate the reliability of the conclusion. There were no changes in the significance of the RR, OS, neutropenia, diarrhea or nausea, which indicated that the conclusion was reliable and stable. Nevertheless, the PFS outcomes clearly changed after excluding the study by Ueno (2016),^[7]^ resulting in a loss of statistical significance between the 2 groups (HR, 0.82; 95%CI 0.56–1.02). This result might be primarily due to the different administration regimens. Based on this discovery, we recommended Ueno (2016)^[7]^ S-1 combination regimen in the following clinical trial or practice regarding to GEM-refractory PC patients. In summary, S-1 combined with both leucovorin and CPT-11 was a good therapeutic choice for GEM-refractory PC patients.

Compared with previous studies, the evidence level of this meta-analysis is level 1, which means the conclusion of this study is convincing and reliable. After searching the 4 biggest medical databases and setting strict inclusion and exclusion criteria, we only included high quality RCTs to achieve comprehensive and credible conclusions, low-quality trails were removed. In addition, we also affiliated our search strategy, which means this study has a good repeatability. Furthermore, we achieved the conclusion that S-1 combined with leucovorin led to longer PFS in the S-1 combination group, the total dose and usage of adjutant leucovorin might affect the final therapeutic efficacy. Thus, administration regimens in Ueno (2016)^[7]^ were recommended in following clinical practice and trials. Concerning the RR, S-1 combined with the irinotecan (CPT-11) regimen resulted in a significantly higher RR than S-1 monotherapy, while the other 2 regimens didn’t provide a significant higher RR. Thus, we hypothesized that S-1 combined with both irinotecan and leucovorin might achieve better therapeutic effects, which provided new combination regime for the following trails.

Several limitations of this study should be noted. First, the sample size was small, and a certain bias might have been introduced due to the small number of studies. Second, all of the trials were conducted in Asian countries (Japan and China) because S-1 first emerged as a potential adjuvant alternative to GEM and was available in several Asian countries but has not been approved in the United States. Thus, the patients included were all Asian, and the conclusions should be applied with caution in the European race. These limitations also suggested an urgent need for more international institutions, particularly in Europe and the United States, to perform research with standardized, multicenter, unbiased methods, and larger sample sizes to confirm the safety and efficacy of the different S-1 combination regimens. Finally, imbalances in the backgrounds of the patients and studies might have affected the assessment of therapeutic efficacy, although subgroup analyses and sensitivity analyses were performed.

This study was conducted at an appropriate time. To the best of our knowledge, no related study has provided the newest information in this research area to date. Although there were some limitations, based on the pooled data, we concluded that the S-1 combination group had a higher response rate and longer PFS than the S-1 monotherapy group. Both groups had a small number of adverse events, with no difference between the groups. The subgroup analysis suggested that S-1 combined with leucovorin regimen could achieve promising efficacy for PFS, whereas S-1 combined with CPT-11 effectively enhanced the response rate. Both combination regimens provided extraordinary results for patients with advanced PC who chose S-1 as the second-line therapy. These 2 combination regimes were practical and crucial in the future clinical practice and clinical trials. The conclusions in this meta-analysis provided guidelines for S-1 rational use. The evidence level of this meta-analysis which included 4 high quality RCTs achieved level 1, to our best known, this is the most convincing, reliable evidence in this area. However, additional high-quality trials are needed to further verify this conclusion. Our findings support the need to compare S-1 monotherapy with combination therapy in concurrent settings in large prospective RCTs.

## Acknowledgment

The authors acknowledged Professor Niu Junqi in the First Hospital of Jilin University for his help.

## Supplementary Material

Supplemental Digital Content
