# Supplementary material for: S-1 monotherapy versus S-1 combination therapy in gemcitabine-refractory advanced pancreatic cancer: A meta-analysis (PRISMA) of randomized control trials
Source: Medicine (Baltimore). 2017 Jul 28;96(30):e7611. doi: 10.1097/MD.0000000000007611 (PMC5627841; doi:10.1097/MD.0000000000007611)
Supplement: Supplemental Digital Content [file medi-96-e7611-s001.docx]

**Appendix 1**

**Search strategy**

Sum=973

Embase: 493

#1 ‘pancreas cancer’/exp

#2 pancrea*

#3 cancer* OR tumour* OR tumor* OR neoplasm* OR carcinoma*

#4 #2 AND #3

#5 neoplasm*, pancrea* OR cancer of pancreas OR cancer*,pancrea* OR cancer* of the pancrea*

#6 #1 OR #4 OR #5

#7 advanced OR unresectable OR borderline resectable OR inoperable

#8 #6 AND #7

#9 ‘S1’/exp

#10 Subfragments, Myosin OR Meromyosin Subfragments OR Heavy Meromyosin Subfragment 1 OR Subfragment-1, Heavy Meromyosin OR Myosin Subfragment 1 OR Subfragment-1, Myosin OR S-1 OR Actin-S1 ATPase OR Myosin S-1 OR Actomyosin Subfragment 1 ATPase OR Heavy Meromyosin Subfragment 2 OR Myosin Subfragment 2 OR Subfragment-2, Myosin OR Myosin S 2 OR Light Meromyosin OR Meromyosin, Light OR Myosin Rod OR Actoheavy Meromyosin OR Heavy Meromyosin

#11 #9 OR #10

#12 #8 and #11

Cochrane: 41

#1 MeSH descriptor: [Pancreatic Neoplasmas] explode all trees

#2 Pancrea* AND (cancer* or tumour* or tumor* or neoplas*)

#3 Neoplasm, Pancreatic

#4 Neoplasm, Pancreas

#5 Neoplasms, Pancreas

#6 Neoplasms, Pancreatic

#7 Cancer of Pancreas

#8 Cancers, Pancreas

#9 Cancer, Pancreatic

#10 Cancers, Pancreatic

#11 Cancer of the Pancreas

#12 #1 OR #2 OR #3 OR #4 OR #5 OR #6 OR #7 OR #8 OR #9 OR #10 OR #11

#13 advanced OR unresectable OR borderline resectable OR inoperable

#14 #12 AND #13

#15 Subfragments, Myosin OR Myosin S-1 OR S-1 OR Myosin S 1 OR Myosin S 2 OR Myosin Rod

#16 #14 AND #15

Pubmed: 276

#1 ((((((((((((((((("Pancreatic Neoplasms"[Mesh]) OR Neoplasm, Pancreatic) OR Pancreatic Neoplasm) OR Pancreas Neoplasms) OR Neoplasm, Pancreas) OR Neoplasms, Pancreas) OR Pancreas Neoplasm) OR Neoplasms, Pancreatic) OR Cancer of Pancreas) OR Pancreas Cancers) OR Pancreas Cancer) OR Cancer, Pancreas) OR Cancers, Pancreas) OR Pancreatic Cancer) OR Cancer, Pancreatic) OR Cancers, Pancreatic) OR Pancreatic Cancers) OR Cancer of the Pancreas

#2 (((advanced) OR unresectable) OR borderline resectable) OR inoperable

#3 #1 AND #2

#4 ((((("Myosin Subfragments"[Mesh]) OR S-1) OR Actin-S1 ATPase) OR Myosin S-1) OR Meromyosin Subfragments) OR Myosin Subfragment 1

#5 #3 AND #4

Web of Science: 692

#1 TS=pancrea*

#2 TS=(cancer*) OR TS=(tumour*) OR TS=(tumor*) OR TS=(neoplasm*) OR TS=(carcinoma*)

#3 #1 AND #2

#4 TS=(advanced) OR TS=(unresectable) OR TS=(borderline resectable) OR TS=(inoperable)

#5 #3 AND #4

#6 TS=(Myosin Subfragments) OR TS=(S-1) OR TS=(Actin-S1 ATPase) OR TS=(Myosin S-1) OR TS=(Meromyosin Subfragments) OR TS=(Myosin Subfragment 1)

#7 #5 AND #6

**Appendix 2**

**Study quality ass****essment graph**

**
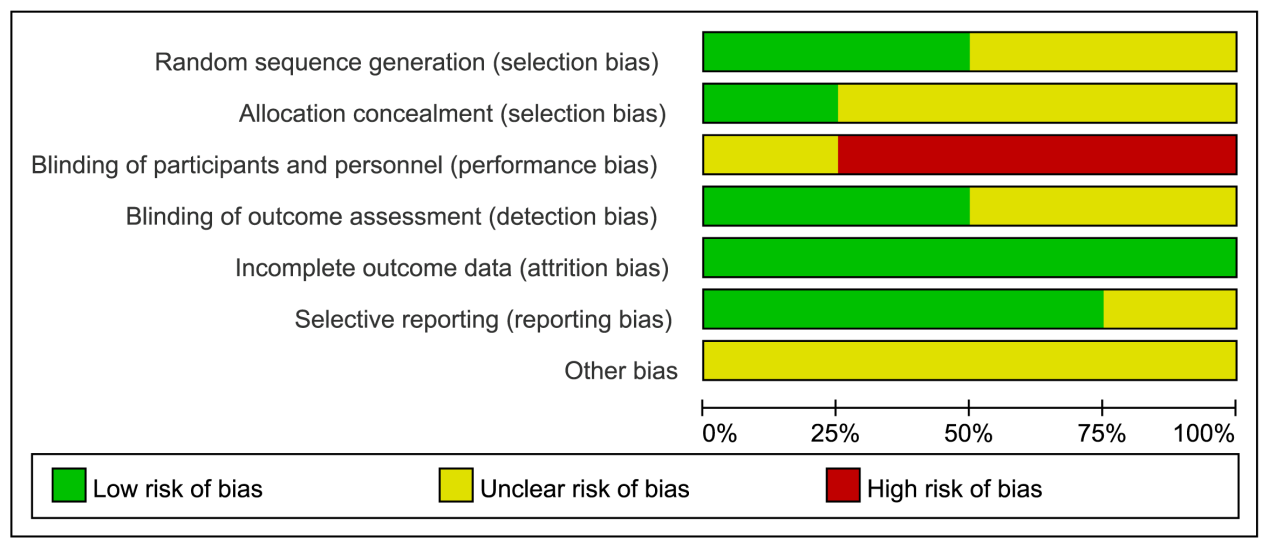
**

**
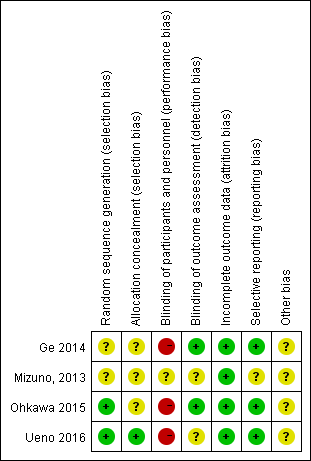
**

**Appendix 3**

**Forest plot of PFS excluding Ueno 2016**

**
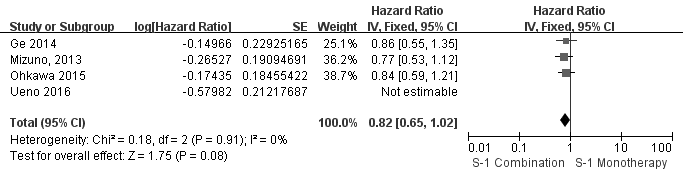
**

**Appendix 4**


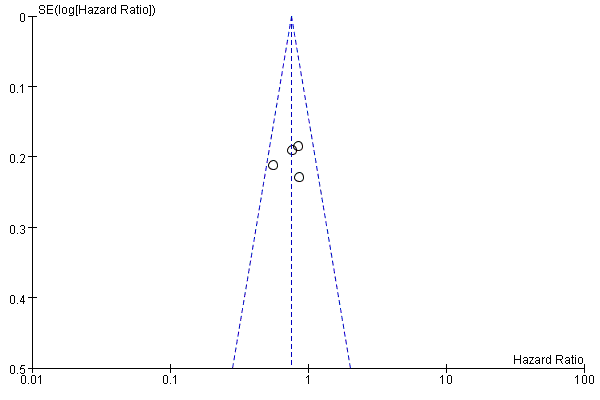


Funnel plots showed there is no publication bias.
